# Molecular Cytology by One-Step Nucleic Acid Amplification (OSNA) Assay of Peritoneal Washings during D2 Gastrectomy in Advanced Gastric Cancer Patients: Preliminary Results

**DOI:** 10.3390/jcm10225230

**Published:** 2021-11-10

**Authors:** Katarzyna Gęca, Karol Rawicz-Pruszyński, Radosław Mlak, Katarzyna Sędłak, Magdalena Skórzewska, Zuzanna Pelc, Teresa Małecka-Massalska, Wojciech P. Polkowski

**Affiliations:** 1 Department of Surgical Oncology, Medical University of Lublin, Radziwiłłowska 13 St., 20-080 Lublin, Poland; kasiaa.geca@gmail.com (K.G.); sedlak.katarz@gmail.com (K.S.); magdalena.skorzewska@umlub.pl (M.S.); zuzanna.torun@gmail.com (Z.P.); wojciech.polkowski@umlub.pl (W.P.P.); 2 Department of Human Physiology, Medical University of Lublin, Radziwiłłowska 11 St., 20-080 Lublin, Poland; radoslawmlak@uml.edu.pl (R.M.); teresa.malecka-massalska@umlub.pl (T.M.-M.)

**Keywords:** advanced gastric cancer, one-step nucleic acid amplification, peritoneal washings

## Abstract

The presence of peritoneal free cancer cells (FCC) in gastric cancer (GC) patients is a poor prognostic factor. D2 gastrectomy may induce exfoliated FCC spread from the primary tumour or involved lymph nodes (LN). Conventional cytology for FCC detection has several limitations, whereas prophylactic use of extensive intraoperative peritoneal lavage (IPL) does not improve survival. A prospective single-arm observational study was conducted to verify whether D2 gastrectomy causes an intraoperative increase of FCC in peritoneal fluid. Twenty-seven GC patients underwent D2 gastrectomy, followed by objective quantitative measurements of CK19 mRNA level reflecting FCC with One-Step Nucleic Acid Amplification (OSNA) assay. The IPL with 3000 mL of saline was performed twice: (1) after gastrectomy with D2 lymphadenectomy and (2) after alimentary tract reconstruction. The IPL samples were analysed by initial cytology and four (1–4) consecutive OSNA assays. Initial OSNA measurement (1) revealed positive results (≥24.6 cCP/μL) in 7 (29.6%) patients. Subsequent OSNA measurements showed a significant decrease in the FCC level after D2 gastrectomy (1 vs. 2; *p* = 0.0012). The first IPL induced a non-significant increase in the FCCs (2 vs. 3, *p* = 0.3300), but the second IPL reversed it to normal levels (3 vs. 4, *p* = 0.0.0574). The OSNA assay indicates a temporal intraoperative increase in the peritoneal FCC in advanced GC patients undergoing D2 gastrectomy. Two consecutive IPLs are necessary to reverse the increase of CK19 mRNA level in peritoneal washings.

## 1. Introduction

The peritoneum is the most common gastric cancer (GC) dissemination site, even if treated with curative intent surgery [1]. Moreover, synchronous peritoneal metastases (PM) are frequently present at the initial GC diagnosis [2]. The prognosis of patients with PM remains dismal [3]. The median survival time is reported to be 3 to 6 months [4]. The survival rates of patients with cytology-positive peritoneal lavage, but without macroscopic peritoneal dissemination (CY1/P0) are reported to be similar to that of patients with overt PM (P1). The 5-year survival rate of these patients is only 2%, with a median survival of 9.2 months [5].

Nevertheless, peritoneal recurrence (PR) has been observed even in T1N0 GC patients [6]. The PM are caused by free cancer cells (FCC) exfoliated from the primary tumour or involved lymph nodes (LN) [7]. Several studies documented that intraoperative FCC spread can occur during gastrectomy for GC [7,8,9] due to tumour manipulation or opening lymphatic channels during dissection of LNs [10]. Therefore, it is crucial to prevent FCC from implanting into the peritoneal lining. An opening of the stomach during gastrectomy may carry a potential risk of peritoneal seeding of FCC upon transluminal communication. However, intraoperative gastric irrigation may minimize the possibility of tumour seeding [11]. Murata et al. reported viable FCC in 23% of patients undergoing gastrectomy that were detected by cytology before anastomosis [12]. One method to remove FCC from the peritoneal cavity is extensive intraoperative peritoneal lavage (EIPL). This approach is based on ‘limiting dilution theory’, which aims to dilute FCC to almost zero. In practice, about ten consecutive washes had been performed with 1 L of physiological saline, which then had to be completely aspirated from the peritoneal cavity [10]. The EIPL plus intraperitoneal chemotherapy (IPC) was shown to improve the 5-year survival in patients with advanced GC and intraperitoneal FCC without overt PM (CY1/P0) [1]. According to the 8th edition of the American Joint Committee on Cancer (AJCC) staging system, positive peritoneal cytology is considered distant metastasis and indicates stage IV disease [5]. Therefore, in many institutions, peritoneal washing cytology is routinely performed during surgery for GC. Despite its low sensitivity ranging from 11 to 80%, cytological evaluation after hematoxylin and eosin (H&E) or Papanicolau staining is still considered the gold standard [13]. The high variability in the sensitivity range implies that cytology may not be regarded as a reliable diagnostic tool and could be the reason for the high PR rate in negative cytology patients [13]. Many methods of molecular cytology have been recently used to detect FCC in peritoneal fluid of GC patients [14]. Some of them DNA CY1 has a great value to detect minimal residual disease of the peritoneum of GC patients [15]. Recently, Sysmex Corp (Kobe, Japan) developed an automated molecular diagnostic assay for intraoperative diagnosis of LN metastasis. So far, this technique has been applied to breast, colorectal, gastric, lung, endometrial, cervical, and prostate cancer [16,17,18,19,20,21,22,23]. The One-Step Nucleic acid Amplification (OSNA) method is based on a precise, efficient, and rapid technique for gene amplification, reverse-transcription loop-mediated isothermal amplification (RT-LAMP) [16,17,24]. The first step of the assay is the homogenization of the LN sample. Then, without RNA purification, the supernatant determines the targeted cytokeratin 19 (CK19) mRNA expression [16,17], which correlates with the size of metastatic foci in the LNs [22] and appears to be equivalent or superior to histopathological examination [24].

Moreover, OSNA may lead to upstaging of GC patients due to increased sensitivity of LN metastases detection [25]. As we previously described, OSNA can be applied with high diagnostic accuracy to detect FCC in intraoperative peritoneal washings of GC patients [26]. Therefore, OSNA may serve as an alternative to conventional cytology. It appears to be a reliable quantitative method for FCC assessment in the peritoneal cavity considering its objectivity and reproducibility [26]. Our previous work showed that in the peritoneal fluid, significantly higher CK19 mRNA values were observed in patients with serosal infiltration and lymph node metastases [26].

For this reason, this prospective single-arm observational study aimed to verify whether D2 gastrectomy in advanced GC patients might cause a significant increase of FCC in the peritoneal washings using the OSNA assay. Additionally, we analysed the impact of intraoperative lavage (IPL) of 6000 (3000 + 3000) mL of saline on the FCC status after surgery.

## 2. Materials and Methods

### 2.1. Study Design

This prospective, single-centre study was conducted between March 2020 and March 2021 in our institution, after obtaining institutional review board approval (Bioethical Committee of Medical University of Lublin, Ethic Code: KE—0254/180/2020). Written informed consent was obtained from the patients in line with the principles outlined in the Declaration of Helsinki. The inclusion criteria were: histologically confirmed, locally advanced gastric adenocarcinoma (cT2-4N1-3M0) scheduled for surgery with curative intent. Pathologic tumour stage was evaluated in the resection specimen, either following neoadjuvant chemotherapy (NAC) or upfront surgery. Modified Becker’s system was used for the evaluation of pathological response [27]. Patients in whom positive cytology was found in samples obtained after laparotomy (CY1) were excluded from further analysis. Ultimately, 27 patients were included in the study.

### 2.2. Intraperitoneal Lavage

**Figure 1:**
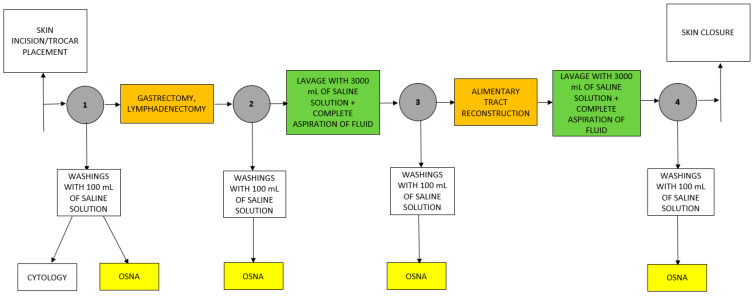
Algorithm of IPL and OSNA assay in GC patients undergoing D2 gastrectomy.

After exploring the abdominal cavity, first washing with 100 mL of saline solution was performed (timepoint #1). The sample obtained was divided into two parts—one intended for cytological examination, the second one for OSNA examination. Then, gastrectomy with lymphadenectomy was performed at the surgeon’s discretion. Afterwards, a second washing with 100 mL of saline was performed (timepoint #2). Next, the first IPL with 3000 mL of saline was conducted. After adequate distribution in the abdominal cavity, the fluid was completely aspirated by suction and discarded. Then, another washing with 100 mL of saline was performed (timepoint #3). The second IPL with 3000 mL of saline was done after alimentary tract reconstruction, followed by the last washing with 100 mL of saline solution (timepoint #4). All washing samples were analysed by OSNA assay (Figure 1). To obtain comparable results for each of the timepoints, all OSNA measurements were performed using 50 mL of peritoneal washings.

### 2.3. OSNA Examination

Peritoneal washing samples intended to OSNA examination were centrifugated for 10 min. at 1500× *g* in order to obtain cellular sediment. The cell pellet was stored in −80 °C until the OSNA examination. As we previously described, peritoneal washings were assessed according to the protocol for OSNA performance [26]. The first step of sample preparation was homogenising cellular sediment using homogenising buffer LYNORHAG, pH 3.5 (Sysmex, Kobe, Japan). During this process, CK-19 mRNA was released from tumour cells. Then, cellular lysate was analysed on an RD-210 gene amplification detector (Sysmex). To perform an RT-LAMP reaction, a ready-to-use LYNOAMP gene amplification reagent kit (Sysmex, Kobe, Japan) was used. The RT-LAMP method measured the time taken to exceed specified threshold turbidity caused by magnesium pyrophosphate, a by-product of the reaction. The change in turbidity correlates with the amount of CK19 mRNA calculated from the value of the standard curve. The limit of detection (LOD) of the RD-210 gene amplification detector was set at 56 cCP/μL. The results below the LOD were calculated based on the standard curve. The cut-off value for distinguishing positive and negative cases was identified as 24.6 cCP/µL. At this cut-off value for peritoneal lavage samples, sensitivity and specificity were 83.3% and 87.8%, respectively [26].

### 2.4. Conventional Cytology

Cytological examination after hematoxylin and eosin (H&E) staining were performed by an experienced cytopathologist from our Hospital Pathology Department.

### 2.5. Statistics

Statistical analysis of the data was performed using the MedCalc software v.15.8 (MedCalc, Ostend, Belgium). Due to the lack of normality of the distribution of the analyzed variables (tested with the D’Agostino–Pearson test), non-parametric tests were used: Wilcoxon test (if two paired groups were compared); ANOVA Friedman test (if more than two paired groups were compared), Mann–Whitney U test (comparisons of two independent groups), or ANOVA Kruskal–Wallis test (comparisons of more than two groups). Moreover, due to the same reason, continuous data are presented as medians with 95% CI. Categorized or dichotomized data are presented by numbers and percentages. Results with *p*-value below 0.05 were considered statistically significant, whereas those between 0.05 and 0.06 as having a tendency toward significance.

## 3. Results

There were 30 patients submitted to the study. Based on positive cytological findings obtained after laparotomy, three were excluded from further analysis (CY1). The characteristics of 27 eligible patients are summarized in Table 1. 

**Figure 2:**
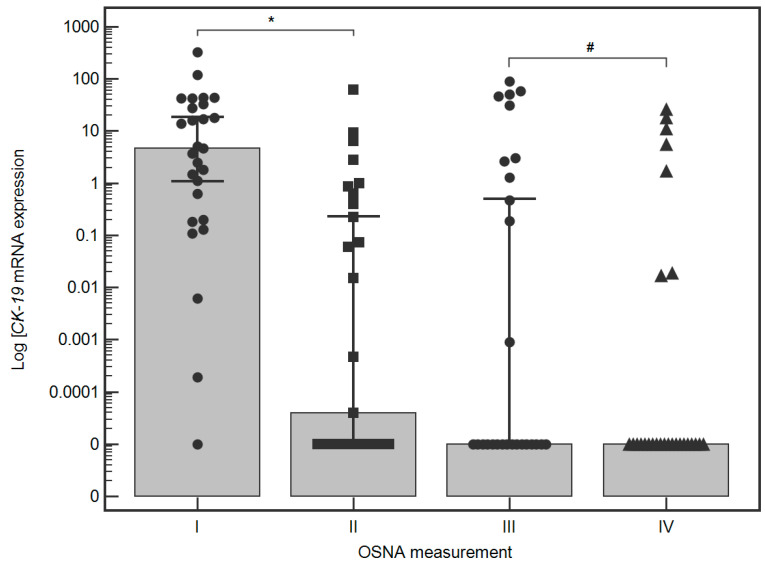
Comparison of four consecutive OSNA measurements performed according to the study protocol. *—*p* < 0.0012; **#**—*p* = 0.0574.

In all four OSNA measurements, there were no statistically significant differences depending on sex, age, Lauren’s type, ypT, ypN, ypM, the use of NAC, PR, or extent of gastrectomy (Supplementary Table S1). Initial OSNA measurement (#1) revealed positive results (≥24.6 cCP/μL) in 7 (29.6%) patients. Subsequent OSNA measurements (#2) revealed a statistically significant decrease in the CK-19 mRNA level after removal of the primary tumour with regional LNs (D2 gastrectomy) (1 vs. 2; medians (95%CI): 4.6 (1.1–18.5) vs. 0.00004 [0–0.2) cCP/μL; *p* = 0.0012). The first IPL induced statistically non-significant increase in the CK-19 mRNA level (#2 vs. #3, medians (95%CI): 0.00004 (0–0.2) vs. 0 (0–0.5) cCP/μL; *p* = 0.3300), but the second IPL reversed it to previously observed levels (#3 vs. #4, medians (95% CI): 0 (0–0.5) vs. 0 (0–0) cCP/μL; *p* = 0.0574). At this point of operation, additional finding was a trend toward temporal increase of CK-19 mRNA level in patients with LN involvement (pN1-3) (#2 vs. #3; medians (95%CI): 0 (0–0.2) vs. 0 (0–42.8) cCP/μL; *p* = 0.0674). Based on our previously established cut-off value, 7 of the patients were positive at timepoint #1. Five of them become negative at subsequent time points (#2, #3, and #4). One of the patients was also positive at timepoint #2. The value of CK-19 mRNA after gastrectomy with lymphadenectomy increased from 33 cCP/µL to 62 cCP/µL. The subsequent measurements (#3 and #4) were negative. One patient remained positive (26.0 cCP/μL) at the end of operation as measured by OSNA (#4) (Figure 2). This patient was negative on cytology at the beginning of surgery (#1) but positive according to OSNA assay (44 cCP/μL). During the operation, three macroscopically visible peritoneal lesions (localised at jejunal mesentery, pancreatic capsule, and mesocolon) have been excised for pathology, which finally revealed PMs (ypT3N1M1[P1b]). Five patients were negative during time points #1 and #2 after the first IPL (time point #3). The application of the second IPL resulted in an adverse finding during measurement #4. Comparisons of the results of subsequent CK-19 mRNA assessments depending on demographic and clinical variables are available in the Supplementary Materials.

## 4. Discussion

This single-institution prospective study indicated that nearly 30% of resectable GC patients were identified as positive for FFC by initial OSNA assay. In contrast, these patients were determined as negative by conventional cytology. We also observed a non-significant increase in the FCC after the initial IPL, which was reversed to previously observed results after the second IPL in pM0(P0) patients. Since EIPL with IPC improved the 5-year OS in C1/P0 patients [1], further studies were conducted to determine the safety and efficacy of extensive peritoneal lavage in GC patients [4,28,29,30] (Table 2). However, none of them indicated a positive effect of the EIPL alone on OS, disease-free survival (DFS), or peritoneal recurrence-free survival. By far, EIPL is effective only in combination with intraperitoneal administration of chemotherapy [1].

Since 3 L of saline might have been sufficient to eliminate FCC [29], we decided to perform two IPL with 3000 mL of saline each instead of 10 lavages with 1000 mL of saline when planning the study. The initial IPL caused a non-significant increase in FCC, which was eliminated after the secondary IPL. Intragastric cancer cell positivity increases by stage and surgical manoeuvre [6]. Conversion from negative intragastric cytology to positive cytology during the operation can be observed, even in early GC patients. Probably, manipulation of the stomach harbouring GC can increase the detachment of FCC into the gastric lumen. These cells can further spread into the peritoneal cavity when the lumen of the stomach is opened during the operation. Thus, the intraluminal FCC can be the source of PR even in serosa-noninfiltrating node-negative GC patients. Our study revealed a statistically significant decrease in the CK-19 mRNA level after excision of the primary tumour with regional LNs (timepoint #1 vs. timepoint #2). We hypothesize that tumour burden per se is a more significant source of cancer cells than surgical manipulation. Therefore, our study included patients with any tumour stage. Han et al. analyzed dissemination of FCC by CEA RT-PCR in an ex vivo surgical specimen study, using clipped versus opened lymphovascular pedicles [8].

The differences in CEA mRNA amplification were more evident when lymphovascular pedicles were closed. In half of the patients, the CEA mRNA levels increased more than twice after the lymphovascular vessels were opened compared to the sealed channels [8]. Technique and extent of LN dissection are crucial aspects of GC surgery. Lymphovascular structures should be well-controlled either with clips or energy-based devices to prevent cancer cell spillage and peritoneal seeding [6]. Ronellenfitsch et al. conducted a study to determine if EIPL eliminates FCC released during gastrectomy and lymphadenectomy for GC [31]. Peritoneal lavage samples were collected in three-time points: after exploration of the peritoneal cavity, after resection and lymphadenectomy, and from the last EIPL performed. Three out of 27 (11%) patients became positive on conventional cytology after surgical procedures.

The study showed that even after the EIPL, these patients were still positive. Additionally, in five more patients with negative cytology before EIPL, FCC were detected after the procedure, suggesting that EIPL itself might lead to the spread of FCC [31]. In contrast to Ronellenfitsch et al., our results showed that in patients who are initially negative with conventional peritoneal cytology, two IPLs with 3000 mL of saline are sufficient to reverse the temporal increase of FCC. Despite its low sensitivity and specificity, conventional cytology has been a gold standard for detecting FCC [13]. Hasbahceci et al. reported that one out of two CY1 patients remained cytologically positive after gastrectomy and 3000 mL saline lavage. There was no conversion of negative to positive cytology result [32]. In our study, the OSNA assay allowed detecting 7 (29.6%) positive patients during the first measurement point. These patients were negative by cytological findings. There are no clear recommendations concerning EIPL and IPC and their clinical benefit in GC patients. Results of the meta-analysis showed that 2- and 5-year OS rates in patients with FCC without macroscopic peritoneal dissemination are increased after IPC [33]. Additionally, IPL further increases these rates and lower the rate of PR [33]. The natural history of patients with GC who have baseline negative peritoneal cytology (CY0) and then undergo multimodality therapy shows subsequent development of peritoneal recurrence in a considerable proportion of patients, even after R0 surgery [34]. Peritoneal recurrence-free survival and OS of patients with amplification of cancer-related genes in peritoneal washings obtained intraoperatively during curative gastrectomy is poor even after a long follow-up [35]. The inability to establish the specificity of the assay shows the preliminary nature of our results. CK19 mRNA might be produced by other cells in the abdomen. Therefore, further studies and respective validation are warranted. Due to the lack of similar studies adopting a designed study protocol (in terms of number and volume of lavages), we decided to test it on a small number of patients to correctly calculate the sample size for further research. At this stage, we obtained meaningful and statistically significant differences, which encouraged us to publish the study’s early findings.

## 5. Conclusions

OSNA assay indicates a temporal intraoperative increase in the peritoneal FCC in advanced GC patients undergoing D2 gastrectomy. Two consecutive IPLs are necessary to reverse the increase of CK19 mRNA level in peritoneal washings. Validation of these preliminary results in a randomised setting is warranted.

## Figures and Tables

**Table 1:** Patients’ characteristics.

Variable	Study Group (*n* = 27)
Sex	
Men	13 (48.1%)
Women	14 (51.9%)
Age	
median (range)	63 (40–80)
<65 years	14 (51.9%)
≥65 years	13 (48.1%)
Lauren’s type	
Intestinal	12 (44.5%)
Mixed	7 (25.9%)
Diffuse	7 (25.9%)
Undetermined	1 (3.7%)
(y)pT	
in situ	3 (11.1%)
1a	2 (7.4%)
1b	4 (14.8%)
2	4 (14.8%)
3	10 (37%)
4a	2 (7.4%)
4b	2 (7.4%)
(y)pN	
0	12 (44.4%)
1	6 (22.2%)
2	4 (14.8%)
3a	2 (7.4%)
3b	3 (11.1%)
(y)pM	
0	26 (96.3%)
1	1 (3.7%)
Neoadjuvant chemotherapy	
Yes	19 (70.4%)
No	8 (29.6%)
No. of neoadjuvant chemotherapy cycles	
median (range)	4 (4–5)
Extent of gastrectomy	
Proximal gastrectomy	5 (18.6%)
Distal gastrectomy	13 (48.1%)
Total gastrectomy	9 (33.3%)

(y)—TNM staging for patients who received neoajuvant chemotherapy.

**Table 2:** Recent studies on extensive intraperitoneal lavage in GC patients.

	CCOG 1102 Trial [29]	SEIPLUS [4]	EXPEL [20]	EIPL vs. Standard Peritoneal Lavage [30]
Author and year	K. Misawa; 2019	J. Guo; 2019	H. K. Yang; 2020	J. Rodriguez-Santiago; 2021
No. of patients randomized	314	662	800	94
No. of patients assigned and analyzed in EIPL group	145	279	396	43
No. of patients assigned and analyzed in surgery group	150	271	401	43
Inclusion criteria	Age 20–80 years Histologically confirmed adenocarcinoma of the stomach cT3-T4 cN-any M0 CY1 or resectable perigastric peritoneal deposits were eligible if treated with curative intent Possibility of achieving R0 or R1 resection (CY1 patients) ECOG performance status of 0 or 1 Adequate organ function Written inform consent	Age 20–80 years ECOG performance status of 0 or 1 Written inform consent cT3-T4 cN-any M0 R0 surgery	Age 21–80 years c T3-T4 M0 Written inform consent	cT3-T4 cN+ M0 P0 Histologically confirmed adenocarcinoma of the stomach R0 surgery
Exclusion criteria	Synchronous or metachronous malignances other tan carcinoma in situ Cancer of remnant stomach Uncontrollable hypertension or diabetes mellitus Systemic administration of corticosteroids	Neoadjuvant chemotherapy or radiotherapy Peritoneal dissemination, distant LN, ovary, liver, lung, brain, or bone metastases Massive ascites or cachexia Current participation in any other clinical trail Severe cardiovascular, respiratory tract, kidney, liver, or psychiatric disease or diabetes Poor compliance	Neoadjuvant chemotherapy or radiotherapy Metachronous cancer Tumour complications that required emergency surgery Metastases or invasion to adjacent structures that precluded a curative resection	Neoadjuvant chemotherapy or radiotherapy if staging laparoscopy was not performed before the treatment to rule out peritoneal carcinomatosis
Type of surgery	Distal or total open gastrectomy with D2 lymphadenectomy	Proximal, distal or total gastrectomy with D2 lymphadenectomy	Open or laparoscopic radical gastrectomy	Total or distal gastrectomy with D2 lymphadenectomy (or D1 in total spleen-preserving gastrectomy)
EIPL group	EIPL using 10 L of saline (1 L for 10 times)	EIPL using 10 L of saline (1 L for 10 times)	EIPL using 10 L of saline (1 L for 10 times)	EIPL using 10 L of saline (1 L for 10 times)
Surgery group	Peritoneal lavage with no more than 3 L of saline before closure of abdomen	Peritoneal lavage with no more than 3 L of saline before closure of abdomen	Peritoneal lavage with no more than 2 L of saline before closure of abdomen	Peritoneal lavage with no more than 2 L of saline before closure of abdomen
Method of peritoneal lavage assessment	Cytology		Cytology	Cytology
Primary endpoint	DFS rate	OS rate	OS rate	OS rate
Secondary endpoints	OS, peritoneal recurrence-free survival, incidence of adverse events	Safety and efficacy of EIPL	DFS, peritoneal recurrence	Incidence of adverse events, type of recurrence
Results	3-year DFS rate: 63.9% in the EIPL group 59.7% in the surgery group 3-year OS rate: 75.0% in the EIPL group 73.7% in the surgery group Peritoneal recurrence-free survival: not significantly different between groups Incidence of adverse events: no intraoperative complications related to EIPL	Mortality: 0% in the EIPL group 1.9% in the surgery group Overall postoperative complication rate: 11.1% in the EIPL group 17.0% in the surgery group Postoperative pain: 10.8% in the EIPL group 17.7% in the surgery group The primary endpoint of 3-year overall survival (OS) is expected to be published after mature follow-up data analysis	OS: 77.0% in the EIPL group 76.7% in the surgery group Adverse events: 60—in the EIPL group 41—in the surgery group The study has been early terminated on the basis of futility. The third interim analysis showed that the predictive probability of OS being significantly higher in the EIPL group was less than 0.5%	3-year OS rate: 62.3% in the EIPL group 64.3% in the surgery group Adverse events: No differences between the groups Location and number of recurrences: No differences between the groups
Conclusions	EIPL did not improve survival or peritoneal recurrence in patients who underwent gastrectomy for advanced GC	EIPL increases the safety of D2 gastrectomy and decrease short-term postoperative complications and wound pain	EIPL + surgery did not have a survival benefit compared with surgery alone and is not recommended for patients undergoing curative gastrectomy for GC	EIPL in patients with locally advanced GC, regardless of peritoneal cytology, has not been effective as prophylaxis of peritoneal recurrence or better survival.

## Data Availability

Not Applicable.

## Supplementary Materials

The following are available online at https://www.mdpi.com/article/10.3390/jcm10225230/s1, Table S1: Comparisons of the results of subsequent CK-19 mRNA assessments depending on demographic and clinical variables.
