# Supplementary material for: Molecular Cytology by One-Step Nucleic Acid Amplification (OSNA) Assay of Peritoneal Washings during D2 Gastrectomy in Advanced Gastric Cancer Patients: Preliminary Results
Source: J Clin Med. 2021 Nov 10;10(22):5230. doi: 10.3390/jcm10225230 (PMC8621409; doi:10.3390/jcm10225230)
Supplement: Supplementary file 1 [file jcm-10-05230-s001.zip › jcm-1337628-supplementary.pdf]

Supplementary Table S1. Comparisons of the results of subsequent CK–19 mRNA assessments depending on demographic and clinical variables.

**Variable**	**Study group (n=27)**							
	**OSNA#1**	***p***	**OSNA#2**	***p***	**OSNA#3**	***p***	**OSNA#4**	***p***
**Sex**								
Men	2.75 [0.18–28]	0.4093	0.0002 [0–0.63]	0.7969	0 [0–1.30]	0.9132	0 [0–0]	0.7291
Women	5.10 [1.17–43.25]		0 [0–1]		0 [0.13.70]		0 [0–44]	
Age								
<65 years	8.75 [0.13–28]	0.6274	0.07 [0–0.86]	0.0716	0 [0–1.30]	0.9563	0 [0–0]	0.5925
≥65 years	4.60 [1.40–43.00]		0 [0.–0.11]		0 [0–2.70]		0 [0–0.44]	
**Lauren`s type**								
Intestinal	4.60 [0.31–34.50]	0.6683	0.63 [0–2.35]	0.5711	0 [0–2.90]	0.7877	0 [0–0.02]	0.9534
Mixed	2.50 [0.37–25]		0.01 [0.0001–0.18]		0 [0–23.25]		0 [0–0.01]	
Diffuse	18 [2.05–41.25]		0 [0–0.04]		0 [0–0.14]		0 [0–4.2]	
**ypT**								
in situ	0.63 [0.29–2.03]	0.7685	0.01 [0.004–0.48]	0.8099	0 [0–0.35]	0.1735	0 [0–0]	0.3911
1a	21.50 [0–43]		0.00002 [0–0.00004]		0.0004 [0–0.0009]		0 [0–0]	
1b	23.85 [2.60–82]		0.23 [0.03–4.80]		0 [0–0]		0 [0–0]	

2	9.40 [1.45–173.50]		0.43 [0.0002–0.93]		17 [2.15–39]		0 [0–9]	
3	10.30 [0.13–33]		0 [0–0.22]		0 [0–51]		0 [0–0.02]	
4a	5.10 [1.27–14.77]		0 [0–2.10]		0.19 [0.05–2]		1.70 [0.42–4.62]	
4b	14 [14–14]		0 [0–0]		0 [0–0]		0 [0–0]	
**ypN**		0.5472		0.1997		0.1657		0.2729
0	10.35 [0.40–38]		0.03 [0–0.74]		0 [0–0.09]		0 [0–0]	
1	3.55 [1.10–16]		0 [0–0.0005]		15.50 [0–51]		0.008 [0–18]	
2	0.95 [0.05–22.40]		1.90 [0.50–4.60]		1.50 [0–46.50]		0 [0–5.50]	
3a	167.55 [5.10–330.00]		0 [0–0]		24.80 [2.60–47]		0.85 [0–1.70]	
3b	21 [14–28]		0.04 [0–0.07]		0 [0–0]		0 [0–0]	
**ypM**		ND		ND		ND		ND
0	4.15 [0.20–28]		0.00002 [0–0.63]		0 [0–2.60]		0 [0–0]	
1	ND		ND		ND		ND	
**Neoadjuvant chemotherapy**		0.2475		0.1281		0.2099		0.3948
Yes	4.60 [0.13–11.77]		0 [0–0.00003]		0.0009 [0–44.90]		0 [0–1.28]	
No	16 [1.20–40.50]		0.06 [0.8]		0 [0–1.02]		0 [0–0]	

**Type of surgery**								
Proximal gastrectomy	0.13 [0.005–4.72]	0.0679	0.63 [0.16–1.34]	0.3028	0 [0–0.32]	0.4163	0 [0–0.004]	0.6720
Distal gastrectomy	2.50 [0.87–43.25]		0.00004 [0–0.14]		0.0009 [0–10]		0 [0–0.004]	
Total gastrectomy	18 [11.42–35.50]		0 [0–1.65]		0 [0–12.89]		0 [0–6.95]	

ND – not determined (only one case in M1 group)
